# Cxcr2 is Required for Osteoclast Regulation, Bone Structure, and Hematological Response During Bone (Re)modeling

**DOI:** 10.1007/s00223-025-01470-x

**Published:** 2026-01-29

**Authors:** Courtney L. Flatt, Allison Fick, Ben James, Finley Hester, Sarah Nano, Adison Steinke, Jun Li, Glen L. Niebur, Laurie E. Littlepage

**Affiliations:** 1 Department of Chemistry and Biochemistry, University of Notre Dame, Notre Dame, IN USA; 2 Harper Cancer Research Institute, University of Notre Dame, 1234 N Notre Dame Avenue, South Bend, IN 46617 USA; 3 Tissue Mechanics Laboratory, Department of Aerospace and Mechanical Engineering, University of Notre Dame, Notre Dame, IN USA; 4 Department of Applied and Computational Mathematics and Statistics, University of Notre Dame, Notre Dame, IN USA

**Keywords:** Bone remodeling, Cxcr2, Osteoclast, Osteoblast, Osteoimmunology

## Abstract

Cxcr2 is a chemokine receptor involved in immune cell trafficking, inflammation, and wound healing that has been implicated in multiple diseases and cancers. However, there is a relative lack of knowledge of the in vivo functions of Cxcr2 in bone cell biology. Here we characterized the skeletal and hematological phenotypes of Cxcr2-deficient mice (Cxcr2 KO) backcrossed onto the FVB/N background. Structural and biomechanical testing demonstrated that Cxcr2 KO caused a significant loss of trabecular and cortical bone volume, altered bone geometry, and an associated loss of bone strength relative to controls. Histological analysis suggests that this is a consequence of increased osteoclast activity in Cxcr2 KO mice, while osteoblasts were not affected. Serum analysis revealed elevated levels of the bone resorption marker CTX-1, with no change in the bone formation marker P1NP, suggesting a shift towards increased osteoclast-mediated bone turnover. While both RANKL and OPG were decreased in Cxcr2 KO bones, the RANKL/OPG ratio was not different compared to WT mice. Additionally, Cxcr2 KO mice displayed systemic immune dysregulation, including elevated serum cytokines and altered hematological parameters, fewer megakaryocytes in bone marrow, higher neutrophil counts in blood, and anisocytosis. Our data point to Cxcr2 as a critical and multifunctional regulator of healthy bone homeostasis, linking immune function with skeletal integrity. This work highlights the Cxcr2 KO model as a valuable system for studying inflammatory bone loss and osteoimmunological interactions.

## Introduction

Cxcr2, a G protein-coupled chemokine receptor, is a critical mediator of immune cell trafficking in physiological and pathological conditions. Cxcr2 is abundantly expressed on myeloid lineage cells and acts as an essential regulator of neutrophil maturation and mobilization from bone marrow [1]. Cxcr2 promotes angiogenesis after binding to its ELR+ ligands that include Cxcl1, Cxcl2, Cxcl3, Cxcl5, Cxcl6, Cxcl7, and Cxcl8 [2]. Cxcr2 signaling also regulates inflammatory responses in allergic asthma, infection, and wound healing [3–5].

Emerging evidence implicates Cxcr2 in the development and metastasis of various cancers, including breast cancer metastasis to bone [6–11]. We found that Cxcr2 signaling activated by the chemokine ligand Cxcl5 is sufficient to promote the colonization of the bone microenvironment by breast cancer cells, and blocking this signaling with a Cxcr2 antagonist decreased cancer proliferation in bone ex vivo [11]. Significantly, in patients, breast cancer cells that have metastasized to bone disrupt the natural homeostasis of bone formation and resorption, resulting in a vicious cycle of tumor growth and bone destruction. While Cxcr2 is implicated in promoting bone colonization, the requirements for Cxcr2 in skeletal development and bone remodeling are less well-defined.

Cxcr2 and its ligands are expressed by cells in the bone microenvironment, including osteoclasts, osteoblasts, and mesenchymal stem cells, suggesting their involvement in the dynamic processes of bone formation and resorption, i.e., bone remodeling. While young growing mice were used for this study, we use “remodeling” to describe both bone modeling during development and subsequent remodeling of mature bone tissue. Both Cxcr2 and Cxcl2 are expressed in the condensing mesenchyme that gives rise to bone and cartilage at mouse embryonic day 11.5, suggesting a role for this pair in bone development [12]. Cxcl1 mediates the recruitment of highly Cxcr2-expressing hematopoietic osteoclast precursor cells to the bone environment [13]. Cxcr2 expression is also upregulated during RANKL-induced osteoclastogenesis in RAW264.7 osteoclast precursor cells, while siCxcr2 treatment suppresses osteoclastogenesis in mouse calvaria [14]. Silencing Cxcr2 signaling in primary osteoblasts also modulates RANKL and OPG expression to suppress osteoclast differentiation, suggesting that Cxcr2 promotes osteoclastogenesis and bone resorption. Interestingly, Cxcr2 signaling also regulates bone regeneration. Cxcr2 signaling via Cxcl1 and Cxcl8 recruits mesenchymal stem cells (MSCs) and promotes their differentiation into chondrocytes to repair cartilage defects [15–17]. These findings highlight an important function for Cxcr2 signaling in orchestrating the delicate balance between bone formation and resorption, which is pivotal for maintaining skeletal integrity.

Cxcr2 signaling may mediate crosstalk between immune cells and bone niche cells that ultimately regulates bone remodeling activity. Consistently, mice lacking a functional Cxcr2 gene (Cxcr2 KO C57BL/6) develop significantly elevated granulopoiesis and defective neutrophil chemotaxis to sites of inflammation [18]. Cxcr2 knockout (KO) mice also have impaired inflammatory responses that include unresolved macrophage accumulation [19] and altered monocyte recruitment during wound healing [3]. Cxcr2 and its ligands are also elevated in early bone fracture repair [20], and Cxcr2 KO mice have delayed bone healing in cranial defects and stress fracture models [21, 22].

While skeletal alterations have been identified in Cxcr2 KO mice [22, 23], the source of these defects—whether through altered myeloid differentiation, immunological signaling, or direct effects on bone remodeling cells—has not been defined. Strain-specific differences in Cxcr2 KO mice have been documented, with varying phenotypes reported across BALB/c, C57BL/6, and FVB/N strains. The original Cxcr2 KO mice were established in the C57BL/6 background and were visually indistinguishable in size and health from their WT counterparts, and no bone phenotype was described [18]. However, mixed background C57BL/6:BALB/c Cxcr2 KO mice had significantly decreased weight gain, increased mortality, and skeletal abnormalities, including rounded facial structure, abnormal stance and gait, and kyphosis [23]. Importantly, we observed early evidence of skeletal phenotypes in Cxcr2 KO mice in the FVB background, which have not been previously described, and differences in other phenotypes, including mortality rate and body fat content, that differ from prior studies in BALB/c, prompting us to investigate it more systematically. Since laboratory mouse strains have genomic differences that alter transgenic phenotypes [24, 25], we investigated the requirement for Cxcr2 in bone health in the FVB/N background to further analyze the immunological and histological bone alterations in Cxcr2-deficient mice. Our results highlight an important role for Cxcr2 as a regulator of osteoclast activity and a determinant of bone homeostasis.

## Materials and methods

### Mice

Mice used in this study were barrier-maintained under specific pathogen-free conditions in the University of Notre Dame Freimann Life Sciences Center. Cxcr2 KO (B6.129S2(C)-Cxcr2^tm1Mwm^/J) mice were acquired from Jackson Laboratories (JAX: 006848) and backcrossed at least 10 generations to the FVB/N background (Charles River, 559NCIFVB). Body composition was measured in 3-, 5-, and 8-week-old male and female Cxcr2 WT, Het, and KO mice with whole-body quantitative magnetic resonance using an EchoMRI Body Composition Analyzer (EchoMRI LLC, Houston, TX). 8-week-old male and female Cxcr2 (FVB/N) mice were euthanized by CO_2_ inhalation, and both hind limbs were dissected. Femurs and tibiae were cleaned of soft tissue, wrapped in gauze saturated with PBS, and stored at -20 °C until biomechanical testing. For histomorphometry, freshly dissected bones were fixed overnight in 4% paraformaldehyde and preserved in 70% ethanol. Animal studies were conducted with approval from the University of Notre Dame Institutional Animal Care and Use Committee (protocols #21-11-6912, #21-12-6936, #21-12-6937, #21-12-6910) and in accordance with guidelines from the National Institutes of Health.

### Pathogen Testing

Fresh stool samples from Cxcr2 WT and KO mice (*n* = 2/genotype) were analyzed for pathogen monitoring using IDEXX BioAnalytics Opti-XXpress/EDx Mouse Prevalent Profile. Pathogens evaluated by PCR included: *Aspiculuris tetraptera*, EDIM, *Entamoeba muris*, *Helicobacter bilis*, *Helicobacter ganmani*, *Helicobacter hepaticus*, *Helicobacter mastomyrinus*, *Helicobacter rodentium*, *Helicobacter* spp., *Helicobacter typhlonius*, MHV, MNV, MPV, MVM, *Myocoptes*, *Radfordia/Myobia*, *Rodentibacter heylii*, *Rodentibacter pneumotropicus*, *Spironucleus muris*, *Syphacia muris*, *Syphacia obvelata*, TMEV.

### Serum P1NP and CTX-1 ELISA Assays

Whole blood was collected from Cxcr2 WT and KO mice by retro-orbital blood draw. Serum was isolated by centrifugation of whole blood and stored at − 20 ℃ until analysis. Levels of CTX-1 and P1NP were measured using the Serum Crosslaps (CTX-1) ELISA (Immunodiagnostic Systems #AC-02F1) and Rat/Mouse P1NP ELISA (Immunodiagnostic Systems #AC-33F1) according to the manufacturer’s protocols.

### Serum Cytokine Analysis

Luminex xMAP technology was used for multiplexed quantification of 32 mouse cytokines and chemokines in serum from Cxcr2 WT and KO mice (*n* = 6/genotype). The multiplexing analysis used the Luminex™ 200 system (Luminex, Austin, TX, USA) by Eve Technologies Corp. (Calgary, Alberta). Thirty-two markers were simultaneously measured using Eve Technologies’ Mouse Cytokine 32-Plex Discovery Assay^®^ (MilliporeSigma, Burlington, Massachusetts, USA) according to the manufacturer’s protocol. The 32-plex consisted of Eotaxin (CCL11), G-CSF, GM-CSF, IFNγ, IL-1α, IL-1β, IL-2, IL-3, IL-4, IL-5, IL-6, IL-7, IL-9, IL-10, IL-12(p40), IL-12(p70), IL-13, IL-15, IL-17, IP-10, KC (CXCL1), LIF, LIX (CXCL5), MCP-1 (CCL2), M-CSF, MIG (CXCL9), MIP-1α (CCL3), MIP-1β (CCL4), MIP-2 (CXCL2), RANTES (CCL5), TNFα, and VEGF. Assay sensitivities of these markers range from 0.3 to 30.6 pg/mL for the 32-plex. Individual analyte sensitivity values are available in the MilliporeSigma MILLIPLEX^®^ MAP protocol.

Nonspecific detection of LIX/Cxcl5 was observed in the 32-Plex Discovery Assay Luminex panel when testing control serum from Cxcl5 WT and KO mice. The Quantikine ELISA kit (R&D #MX000), which has a higher sensitivity for Mouse LIX, was therefore validated with Cxcl5 WT and KO serum controls and used to determine CXCL5 levels in Cxcr2 WT and KO serum according to the manufacturer’s protocol. G-CSF levels in Cxcr2 WT and KO serum were validated by Quantikine ELISA kit (R&D #MCS00) according to the manufacturer’s instructions. The assay sensitivity for GCSF is reported to be < 5 pg/mL in the ELISA and 2.7 pg/mL in the Milliplex assay.

### Bone Conditioned Media RANKL and OPG ELISA Assays

RANKL and OPG levels in Cxcr2 WT and KO serum were quantified by Duoset ELISA kit (R&D #DY462 and #DY469) according to the manufacturer’s instructions. The assayed conditioned media samples were collected from ex vivo bone cultures 3–5 days after isolation of femurs and tibiae from Cxcr2 WT and KO bones and preparation for culture and daily partial media change [11]. The assay sensitivity for RANKL and OPG was > 62.5 pg/mL. In the Cxcr2 FVB mouse, the serum RANKL concentration was below the detection limit.

### Immune Cell Profiling by Blood CBC with Differential via Heska Hematology Analyzer

The circulating immune cell profile of Cxcr2 WT and KO mice (*n* = 4 each) was determined by Element HT5 Veterinary Hematology Analyzer (Heska Corporation, Loveland, CO). Blood was collected via cardiac puncture in 0.5 M EDTA to prevent clotting. After collection, 15 µL of each sample was immediately measured. The number and percentage of neutrophils, lymphocytes, monocytes, eosinophils, and basophils were determined, as well as red blood cell and platelet parameters.

### Micro-computed Tomography (µCT)

Tibiae and femurs from 8-week-old Cxcr2 WT, Het, and KO mice were scanned and analyzed on a Scanco Medical µCT-80 system (Scanco Medical, Brüttisellen, Switzerland). Images were acquired at 70 kVp, 114 µA at 10-µm resolution, reconstructed with beam hardening corrections, and calibrated for bone mineral density. Images were smoothed with a Gaussian filter (variance = 1 and support = 2) with a 1324 mg/cc threshold used to segment bone. The cortices were analyzed in a 0.4 mm region beginning 6 mm distal to the femoral head. The Scanco midshaft evaluation protocol was used to measure areal properties of the cortex.

For trabecular bone analysis, the volume of interest in the proximal femur started in the femoral neck (considered the region where there was a continuous medullary area distal to the femoral head and the greater trochanter) and extended 1.4 mm distally. The outer cortex of the bone was contoured automatically, and the outer 20 voxels were peeled off to remove the cortex. A 20 voxel size was chosen because the cortical thickness was determined to be between 150 and 200 microns. The Scanco trabecular morphology protocol was used to quantify trabecular architecture and density.

### Bone Strength Testing

Bone strength and stiffness were analyzed by three-point bending tests. Dissected whole femurs from 8-week-old male and female Cxcr2 WT, Het, and KO mice were tested. The posterior aspect of each femur was placed on supports of a three-point bending fixture with a 5 mm span. The proximal aspect of the femoral condyles was in contact with one support to achieve consistent placement. Loading was perpendicular to the longitudinal axis of the diaphysis at a constant displacement rate of 10 mm/min using a 25 lb. load cell. The displacement and load were recorded, and the maximum load was determined. The bending stiffness was calculated as the slope of the load-displacement curve from the start of the linear portion to 20% of the maximum load.

### Quantification of Bone Mineral Content

Bone was ashed to assess mineral content. Excised femurs were weighed wet, then dried for 24 h at 100 °C and weighed to determine dry bone weight, then ashed at 600 °C for 24 h. Bone mineral content was quantified as the percent of ash weight/dry bone weight.

### Bone Processing, Histology Staining, and Immunohistochemistry

After µCT scanning, bones were decalcified in 10% weight/volume EDTA (pH 7.6) at 4 °C for 3 weeks, infused with paraffin in an automated tissue processor (Leica TP1020), embedded into paraffin blocks, and sectioned (4–6 μm) onto slides for histological analysis. Slides were coated with VectaBond Reagent (Vector Laboratories #SP-1800-7) according to the manufacturer’s instructions.

Hematoxylin and Eosin (H&E) staining (Leica Biosystems, # 3801571 and #3801606) and TRAP staining (Sigma-Aldrich #387A-1KT) were done according to the manufacturer’s instructions. TRAP-stained bone sections were counterstained with methyl green, and slides were scanned with Aperio ScanScope. To determine osteoclast surface area (%Oc.Pm/B.Pm), the length of trabecular bone covered by individual osteoclasts was manually measured using NIH ImageJ. The trabecular bone surface area and osteoclast surface area (sum of individual osteoclast lengths) were measured in a 1.4 mm^2^ field of view beginning at the tibial growth plate and extending 1 mm distally.

Immunohistochemistry staining utilized ABC kit and DAB kit (Vector Laboratories, Burlingame, California) according to the manufacturer’s instructions. Sodium citrate antigen retrieval was conducted in a 60 °C water bath for 10 min. Primary antibodies used were goat polyclonal against Alkaline phosphatase (1:1000, R&D #AF2910) or rabbit monoclonal against CD61/integrin β3 (1:500, Cell Signaling #13166). The same concentrations of goat or rabbit IgG were used to replace the primary antibody as a negative control. The slides were counterstained with Hematoxylin QS (Vector Laboratories) and imaged at 50X magnification on Zeiss Observer A1 microscope (Carl Zeiss Microscopy, Germany). For quantification, alkaline phosphatase-positive osteoblast perimeter was measured in ImageJ in the trabecular bone region beginning at the tibial growth plate and extending 1 mm distally. Megakaryocytes were identified by CD61 staining, and the average number of cells was determined by manually counting three 500 µm^2^ fields of view in the metaphysis (beneath the growth plate) and in the diaphysis per bone sample.

### Statistical Analysis

Statistical analysis was completed primarily using GraphPad Prism software (Version 10.0). To compare overall survival, a log-rank test was used for the comparison of survival time between each pair of groups. Deaths were censored in the case of planned euthanasia or for subjects used in experimental procedures. Deaths occurring before genotypic sampling (around 3 weeks old) were not included in the analysis. The statistical analysis of cytokine expression was completed using the R programming language.

Data from male and female WT, Het, and KO animals were compared by two-way ANOVA using Tukey’s multiple comparisons test for post-hoc analysis. Direct comparisons between WT and KO samples, including serum bone turnover markers and histological analyses, were compared by Welch’s *t*-test or Mann-Whitney test and are specified in figure legends.

## Results

### Developmental Defects in the Cxcr2 KO FVB/N Mice Cause Decreased Survival, Body Weight, and Body Fat 

**Fig. 1:**
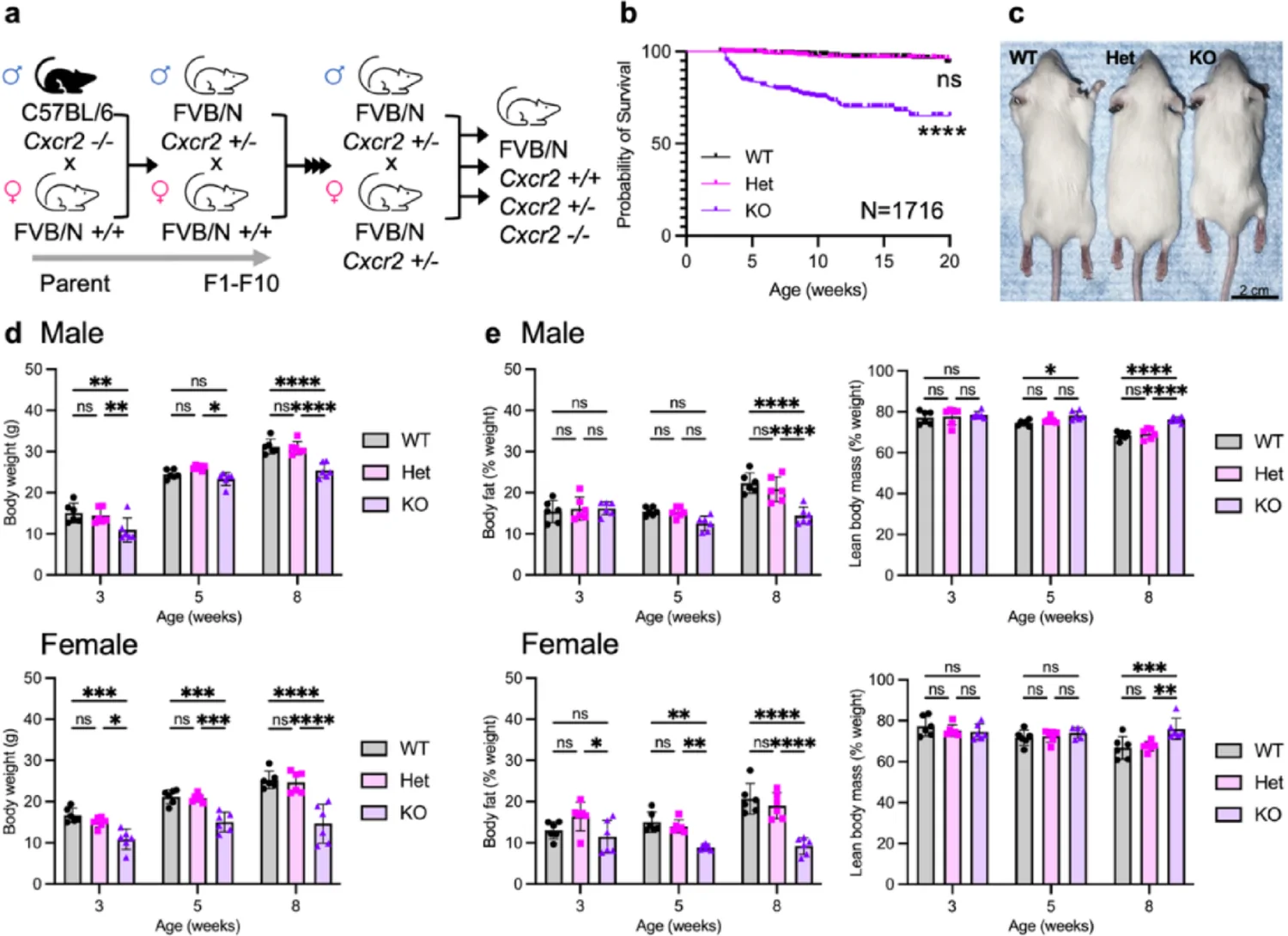
Breeding strategy, survival, and body composition in Cxcr2 KO mice. **a** Breeding scheme to generate Cxcr2 KO mice on FVB/N background. **b** Kaplan-Meier plot showing survival of healthy Cxcr2 WT, Het, and KO mice. Significance was determined using the log-rank (Mantel-Cox) test. **c** Representative photograph of female Cxcr2 WT, Het, and KO FVB/N mice. **d** Body weight and **e** EchoMRI body composition analysis of body fat and lean body mass in 3-, 5-, and 8-week-old male (top) and female (bottom) Cxcr2 WT, Het, and KO mice. Statistical analysis by ANOVA. Lines indicate mean with standard deviation. Significance levels for differences are indicated: * *p* < 0.05, **p < 0.01, ***p < 0.001, ****p < 0.0001, ns: not significant (*p* ≥ 0.05). *N* = 1716 mice (469 WT, 908 Het, 339 KO) for survival analysis; *N* = 6 mice per group for body composition analysis

We backcrossed transgenic *Cxcr2 -/-* mice from the C57BL/6 background (Cxcr2 KO (C57BL/6)) onto the FVB/N background for more than 10 generations (Fig. 1a). After backcrossing the mice, we observed developmental defects as well as significantly decreased survival of Cxcr2 KO FVB mice compared to Cxcr2 wildtype (WT) and heterozygous (Het) mice (Fig. 1b). Cxcr2 KO animals were often runted with rounded facial features (Fig. 1c).

We measured body weight in 3-, 5-, and 8-week-old virgin male and female mice (Fig. 1d; *n* = 6/genotype). In male animals, the mean body weight in 3-week-old Cxcr2 KO mice was only 10.98 g, compared to 15.10 g in 3-week-old WT mice (*P* = 0.0011). Body weight difference was not statistically significant in 5-week-old male Cxcr2 KO compared to WT mice, while 8-week-old Cxcr2 KOs were significantly smaller, with an average weight of 25.45 g in Cxcr2 KO versus 31.19 g in WT mice, respectively, an 18% reduction in normal body weight (*P* < 0.0001).

The body weight differences were more pronounced in female KO mice than in males. The Cxcr2 KO mice were notably smaller than WT and Het littermates from birth and had delayed growth up to 8 weeks. Significantly, the mean body weight in 3-week-old Cxcr2 KO mice was only 10.85 g, compared to 16.67 g in 3-week-old WT mice. At 5 weeks, Cxcr2 KO body weight was 15.07 g, on average, versus 21.18 g for WT, while at 8 weeks old the average weight was 14.64 g versus 25.28 g in Cxcr2 KO and WT mice, respectively. Thus, by 8 weeks of age, female Cxcr2 KO mice were only 58% of the normal weight of their WT littermates (*P* < 0.0001). Therefore, Cxcr2 deficiency significantly decreased the ability to grow and thrive in these mice.

To assess body fat and lean content, we used EchoMRI body composition analysis (EchoMRI LLC, Houston, TX) in 3-, 5-, and 8-week-old male and female mice (Fig. 1e; *n* = 6/genotype). Younger male mice did not significantly differ in fat or lean content, while in 8-week-old males, the fat content decreased by 35% in Cxcr2 KO mice compared to WT mice, with a corresponding increase in lean body content. Like body weight, the body composition differences were more prominent in female mice. At 3 weeks, the body fat content (as a percentage of body weight) in female Cxcr2 KO mice was not significantly different from WT. However, fat content in the KO mice markedly decreased as they aged, with a 40% reduction compared to WT by 5 weeks and a 55% decrease in body fat compared to WT at 8 weeks.

Accordingly, lean content did not differ significantly in 3- or 5-week mice but increased 14% in 8-week-old KO mice versus WT littermates.

### Increased Serum Cytokines and Bone Resorption Marker Expression in Cxcr2 KO Mice

**Fig. 2:**
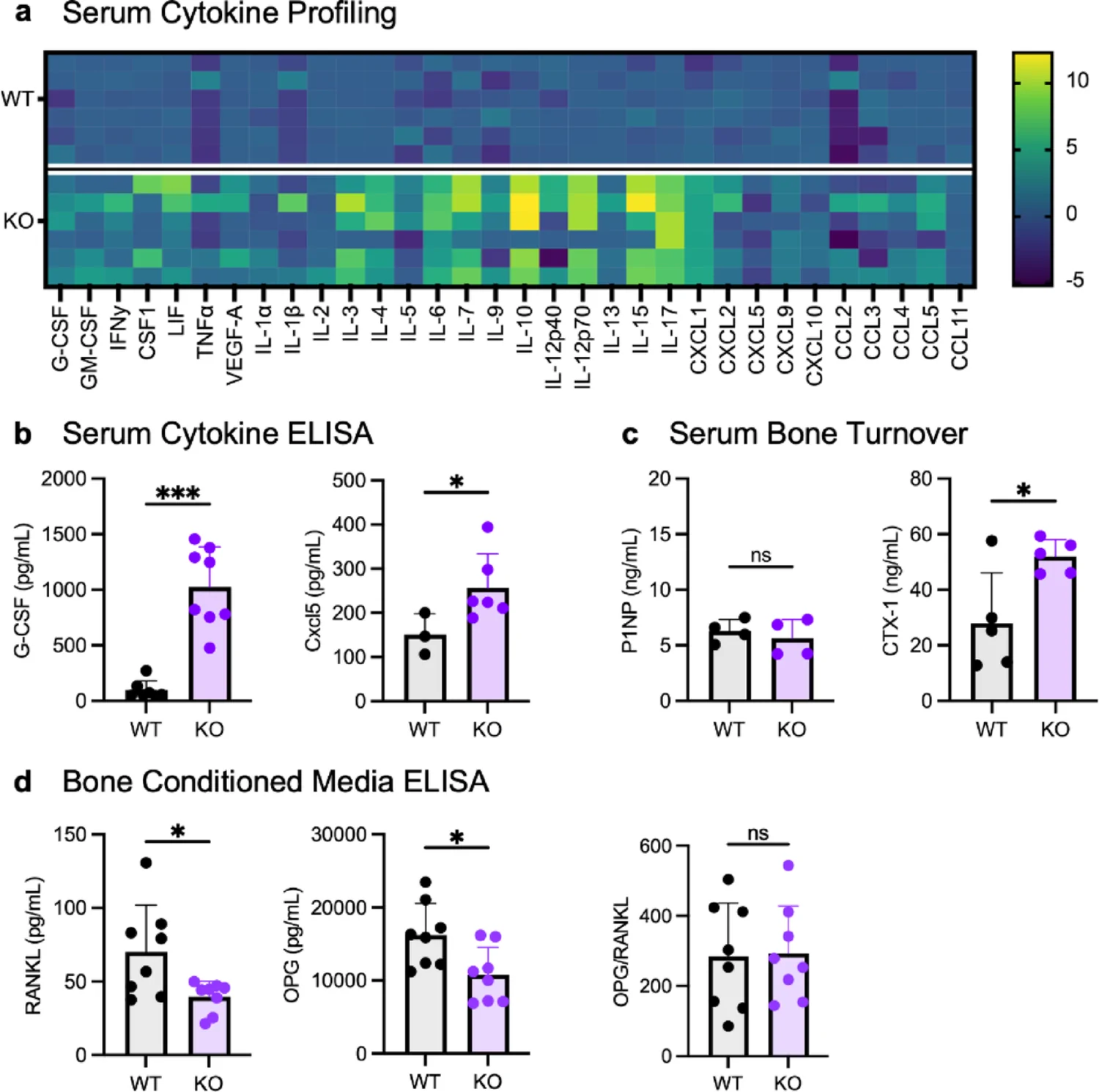
Cxcr2 deletion expands the detected pro-inflammatory cytokines and alters systemic bone turnover. **a** Log-2 transformed fold change in cytokine expression (KO/WT) in Cxcr2 WT versus KO serum, quantified by Eve Technologies 32-Plex Cytokine Array. *N* = 6 mice per genotype. **b** Serum concentration of G-CSF and CXCL5 in Cxcr2 WT and KO mice. *N* = 3–8 mice per genotype. **c** Serum concentration of osteoblast and osteoclast activity markers P1NP and CTX-1 in Cxcr2 WT and KO mice. *N* = 4–5 mice per genotype. **d** Concentration of RANKL and OPG and OPG/RANKL ratio in Cxcr2 WT and KO bone conditioned media. *N* = 8 samples per group. Error bars represent standard deviation. Analysis of Cxcr2 WT versus KO using Welch’s *t-*test. Significance levels for differences are indicated: * *p* < 0.05, **p < 0.01, ***p < 0.001, ns: not significant (*p* ≥ 0.05). N = 4–6 mice per group

Given the considerable structural phenotypic abnormalities of the Cxcr2 KO mice, we began to assess the differences in cytokine expression and bone development. We profiled the serum from WT and Cxcr2 KO mice by cytokine array (32-Plex Cytokine Array, Eve Technologies) to assess the consequence of Cxcr2 deficiency on systemic cytokine signaling (Fig. 2a). Cxcr2 KO serum contained significant elevation in multiple cytokines (*P* < 0.0001), including G-CSF, M-CSF, GM-CSF, VEGF, TNFα, IFNγ, LIF, IL-3, IL-4, IL-6, IL-7, IL-10, IL-12p70, IL-13, IL-15, IL-17, CXCL1, and CXCL9. We also measured cytokine expression of G-CSF and CXCL5 by ELISA (Fig. 2b, left). Increased G-CSF levels in Cxcr2 KO serum were validated by ELISA (*P* = 0.0003). Due to the nonspecific detection of LIX/Cxcl5 in the Luminex panel, we also quantified CXCL5 levels in WT and KO serum by ELISA (Fig. 2b, right). CXCL5 was significantly elevated in Cxcr2 KO mice (*P* = 0.0410), which is consistent with a lack of receptor binding, which elevated chemokine levels in circulation. These data support a role for Cxcr2 in the suppression of cytokines in serum. These factors are candidate cytokine regulators of bone remodeling that crosstalk with Cxcr2 signaling. We assessed the systemic levels of bone turnover markers in Cxcr2 KO mice by serum analysis. Type I collagen is the main protein of the bone matrix. During bone formation, type I collagen is cleaved into procollagen type 1 N-terminal propeptide (P1NP) and C-terminal (P1CP). Elevated serum P1NP levels indicate increased bone formation. In contrast, the C-terminal telopeptide of type 1 collagenase (CTX-1), an accepted clinical marker for fracture risk, is a byproduct of osteoclast activity that increases during bone resorption [26].

Because P1NP and CTX-1 are used as standard bone formation and resorption markers, respectively, for assessing bone remodeling activity, we analyzed P1NP and CTX-1 levels by ELISA in serum (Fig. 2c). While P1NP levels were not different in WT and KO mice, CTX-1 was significantly elevated in KO serum, indicating excess bone resorption with Cxcr2 deletion. Specifically, the mean CTX-1 level measured in WT serum was 27.96 ng/mL, compared to 52.04 ng/mL in KO serum (*P* = 0.0384).

We also measured the levels of RANKL and OPG, critical regulators of osteoclastogenesis, in conditioned media collected in ex vivo cultures from Cxcr2 WT or KO tibia and femur bones. RANKL and OPG levels then were used to calculate the localized RANKL/OPG ratio, an indicator of bone resorption index (Fig. 2d). Both RANKL and OPG decreased significantly in Cxcr2 KO bone versus WT bone. The mean RANKL concentration was 70.32 ± 31.66 pg/mL in WT and 39.74 ± 10.57 pg/mL in KO bone conditioned media (*P* = 0.0303). OPG concentration was 16,215.57 ± 4349.43 pg/mL in WT and 10,784.21 ± 3763.25 pg/mL in KO bone conditioned media (*P* = 0.0185). This is a reduction by 33.49% in RANKL and 43.4% in OPG concentration in Cxcr2 KO bone compared to WT. However, the OPG/RANKL ratio did not differ between Cxcr2 WT and KO bone. Thus, we focused on assessing the role of Cxcr2 in bone resorption and bone strength.

### Decreased Cortical Bone Area and Bone Strength in Femurs of Cxcr2 KO Mice

**Fig. 3:**
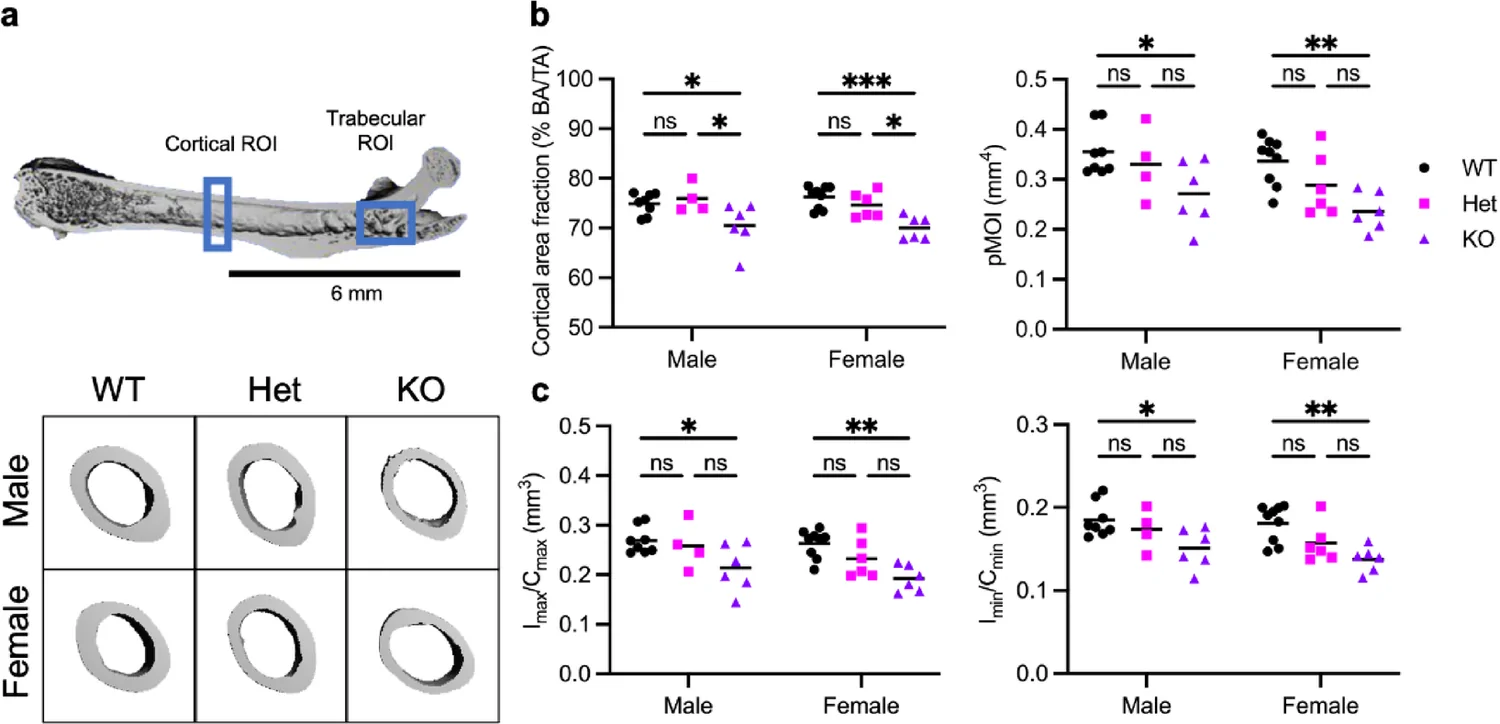
Decreased cortical bone by µCT. **a** Regions of interest for microCT analysis for trabecular and cortical analysis (top) and representative 3D reconstructions of cortical bone at the femur midshaft (bottom). **b** µCT quantification of cortical bone area fraction (left) and polar moment of inertia (pMOI) (right). **c** µCT quantification of major and minor section moduli (I_max_/C_max_ and I_min_/C_min_). Lines indicate the mean. Significance levels for differences (ANOVA) are indicated: * *p* < 0.05, **p < 0.01, ***p < 0.001, ns: not significant (*p* ≥ 0.05). *N* = 4–6 mice per group

To examine the effects of Cxcr2 deficiency on bone structure, we quantitatively evaluated three-dimensional cortical bone in femurs of 8-week-old male and female Cxcr2 KO mice using µCT analysis (Fig. 3a). We quantified cortical bone area, polar moment of inertia, and section moduli (Fig. 3b and c).

Cortical bone area did not vary by sex but decreased significantly by deletion of Cxcr2 (*P* < 0.0001). The mean cortical area fraction (% BA/TA) was 74.72% and 75.24% in WT males and females and 75.80% and 73.65% in Het males and females, respectively. Meanwhile, Cxcr2 KO cortical % BA/TA decreased to 70.22% in males and 69.41% in females, which is a 7% reduction in cortical bone area in KO mice compared to WT (Fig. 3b, left).

Cortical polar moment of inertia (pMOI), which is a measure of the distribution of material and directly affects torsional stiffness, decreased significantly in Cxcr2 KO bones regardless of sex (Fig. 3b, right). Specifically, the mean pMOI decreased by 23.6% in male and 30.1% in female KO femurs compared to WT bones.

Section moduli (I_max_/C_max_ and I_min_/C_min_), which are indicators of bone resistance to bending, were similarly significantly decreased in KO mice compared to WT mice (Fig. 3c; *P* = 0.0002) and were independent of sex. The major axis section modulus (I_max_/c_max_) was 0.269 ± 0.027 and 0.263 ± 0.028 in WT males and females and 0.259 ± 0.048 and 0.232 ± 0.039 in Het males and females, respectively. Meanwhile, Cxcr2 KO exhibited a section modulus of just 0.214 ± 0.047 in males and 0.192 ± 0.027 in females. The minor axis section modulus (I_min_/c_min_) was 0.185 ± 0.021 and 0.181 ± 0.022 in WT males and females and 0.173 ± 0.025 and 0.157 ± 0.024 in Het males and females, respectively. I_min_/c_min_ decreased to 0.151 ± 0.024 in KO males and 0.138 ± 0.015 in KO females. This is consistent with the changes in the pMOI and cross-sectional area.

### Decreased Bending Strength of Cxcr2 KO Bone

**Fig. 4:**
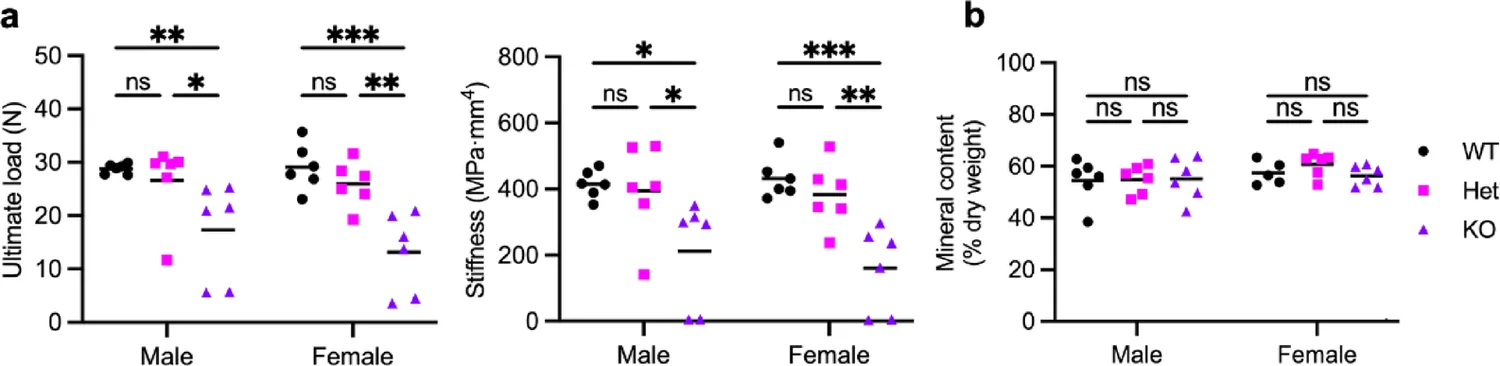
Reduced bone strength in Cxcr2 KO mice. **a** Three-point bending test. Femur bending strength was measured by (Left) ultimate load and (Right) stiffness. **b** Bone mineral content by percent ash weight of dry bone weight determined after bone ashing. Lines indicate mean. Significance levels for differences (ANOVA) are indicated: * *p* < 0.05, **p < 0.01, ***p < 0.001, ns: not significant (*p* ≥ 0.05). *N* = 6 mice per group

We next evaluated the consequence of these geometric changes on bone mechanical properties by measuring three-point bending stiffness and strength, and mineral content. First, we conducted three-point bending tests of femurs from 8-week-old male and female Cxcr2 KO mice to assess bone strength and stiffness (Fig. 4). While there were no sex-dependent differences, Cxcr2 KO bones broke at lower ultimate loads and were less stiff than WT and Het femurs in both males and females (Fig. 4a). The ultimate breaking load of male Cxcr2 KO femurs was 19.6 ± 5.8 N compared to 29.1 ± 1.8 N for Het mice (*P* = 0.0009) and 28.8 ± 0.9 N for WT mice (*P* = 0.001), representing a 40% reduction. Similarly, the ultimate bending force in female Cxcr2 KO mice was 17.8 ± 2.7 N compared to 27.0 ± 2.8 N for Het mice (*P* = 0.0008) and 29.1 ± 4.4 N for WT mice (*P* = 0.0001), a 55% reduction in KO compared to WT bones.

The bending stiffness (i.e., modulus multiplied by moment of inertia) of male KO mice was 260.2 ± 87.7 MPa·mm^4^ compared to 415.4 ± 41.9 MPa·mm^4^ for WT mice (*P* = 0.004) and 433.3 ± 70.4 MPa·mm^4^ for Het mice (*P* = 0.001). Similarly, the bending stiffness of female Cxcr2 KO mice was 227.5 ± 50.9 MPa·mm^4^ compared to 415.4 ± 41.9 MPa·mm^4^ for WT mice (*P* = 0.00009) and 416.3 ± 69.2 MPa·mm^4^ for Het mice (*P* = 0.0002). This signifies a 49% and 63% reduction in bone stiffness in male and in female Cxcr2 KO mice, respectively, compared to WT mice.

Bone bending stiffness is dependent both on bone geometry and bone tissue modulus, which in turn depends on the mineral content [27, 28]. To test if the observed mechanical weakness in KO bones is due to differences in bone mineral content, we dried and ashed 8-week-old Cxcr2 WT, Het, and KO femurs. The bones did not differ significantly in total weight, water content, or mineral content by ash weight (data not shown and Fig. 4b). The mean percent ash weight in male Cxcr2 KO mice was 55.21 ± 8.27%, compared to 54.42 ± 8.49% in male WT and 54.81 ± 5.50% in male Het mice. Similarly, the mean percent ash weight in female Cxcr2 KO mice was 56.27 ± 3.99%, compared to 64.21 ± 17.18% in female WT and 60.72 ± 4.51% in female Het mice. Thus, it is likely that the altered mechanical behavior of Cxcr2-deficient femurs is attributable to these changes in the cross-sectional geometry. However, the nonuniform geometry and size difference of the bones hinder accurate comparison of mechanical properties [29].

### The Trabecular Bone Architecture was Degraded and Volume Fraction Decreased in Cxcr2 KO Mice

**Fig. 5:**
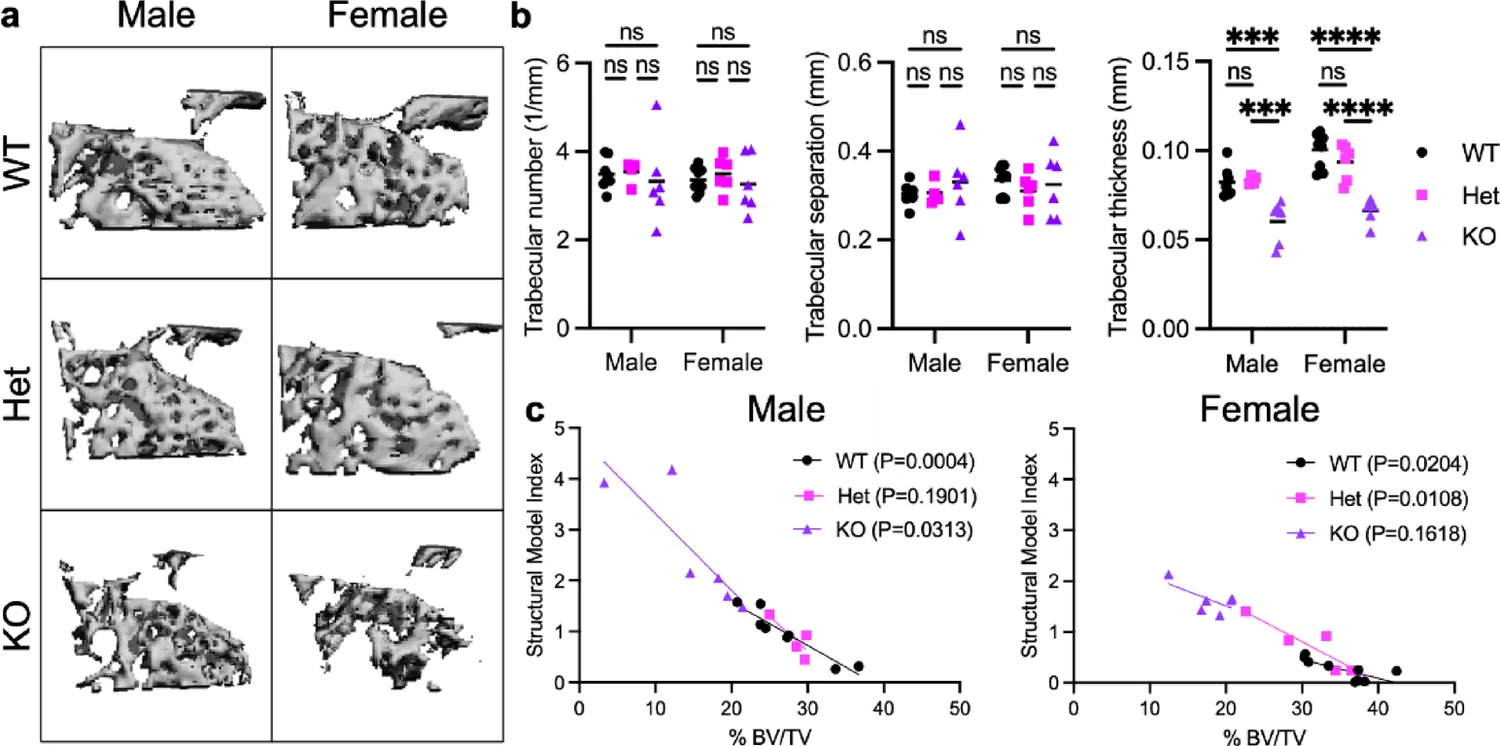
Decreased trabecular bone by µCT. **a** Representative 3D reconstructions of trabecular bone in the proximal femur of 8-week-old male and female Cxcr2 WT, Het, and KO mice. **b** Quantification of trabecular bone parameters. Lines indicate mean. Significance levels for differences (ANOVA) are indicated: * *p* < 0.05, **p < 0.01, ***p < 0.001, ns: not significant (*p* ≥ 0.05). **c** Correlation of trabecular BV/TV and structural model index is sex- and genotype-dependent. Statistical analysis by simple linear regression. *N* = 4–6 mice per group

We next used µCT to evaluate trabecular architecture in the proximal femur (Figs. 3a and 5a). While the trabecular number and spacing were unchanged based on Cxcr2 expression, the average trabecular thickness decreased 26% in male and 34% in female Cxcr2 KO mice compared to in WT mice (Fig. 5b, *P* < 0.0001).

Bone volume fraction (BV/TV) decreased significantly in Cxcr2 KO mice (Fig. 5c). BV/TV was 27.2% and 35.3% in WT males and females and 28.2% and 32.0% in Het males and females, respectively. In contrast, Cxcr2 KO BV/TV decreased to 14.9% in males (45% decline) and 17.9% in females (49% decline) (*P* < 0.0001).

The structural model index (SMI) is a measure of the trabecular structure that distinguishes more robust plate-like from more damage-prone rod-like morphologies [30]. The SMI was sex-dependent (*P* = 0.0027) and increased significantly in Cxcr2-deficient trabecular bone (*P* < 0.0001), with a greater difference in male mice (Fig. 5c). In males, SMI was 0.961 ± 0.488 in WT and 0.854 ± 0.375 in Het, while Cxcr2 KO SMI increased to 2.580 ± 1.169 (*P* < 0.0001). In females, SMI was 0.264 ± 0.207 in WT and 0.650 ± 0.483 in Het mice, with Cxcr2 KO SMI increased to 1.634 ± 0.278 (*P* < 0.0001), indicating a more rod-like trabecular architecture in Cxcr2 KO compared to in WT mice. While SMI is correlated with BV/TV within anatomic sites, we analyzed the data by linear regression. SMI and BV/TV were significantly correlated in WT mice (both male and female) and in male Cxcr2 KO mice (Fig. 5c). We compared the slope of SMI versus BV/TV within genotypes by sex, and only WT males versus females significantly differed (*P* = 0.0146), while Het and KO mice did not show dependence on sex. Within sexes, the correlation of SMI and BV/TV did not differ significantly by genotype. Together, the decreased Tr.Th and increased Tr.Sp and SMI in Cxcr2 KO bone demonstrate the deteriorated trabecular architecture in Cxcr2 KO mice.

### Cxcr2 Deletion Increases Osteoclast Surface Area

**Fig. 6:**
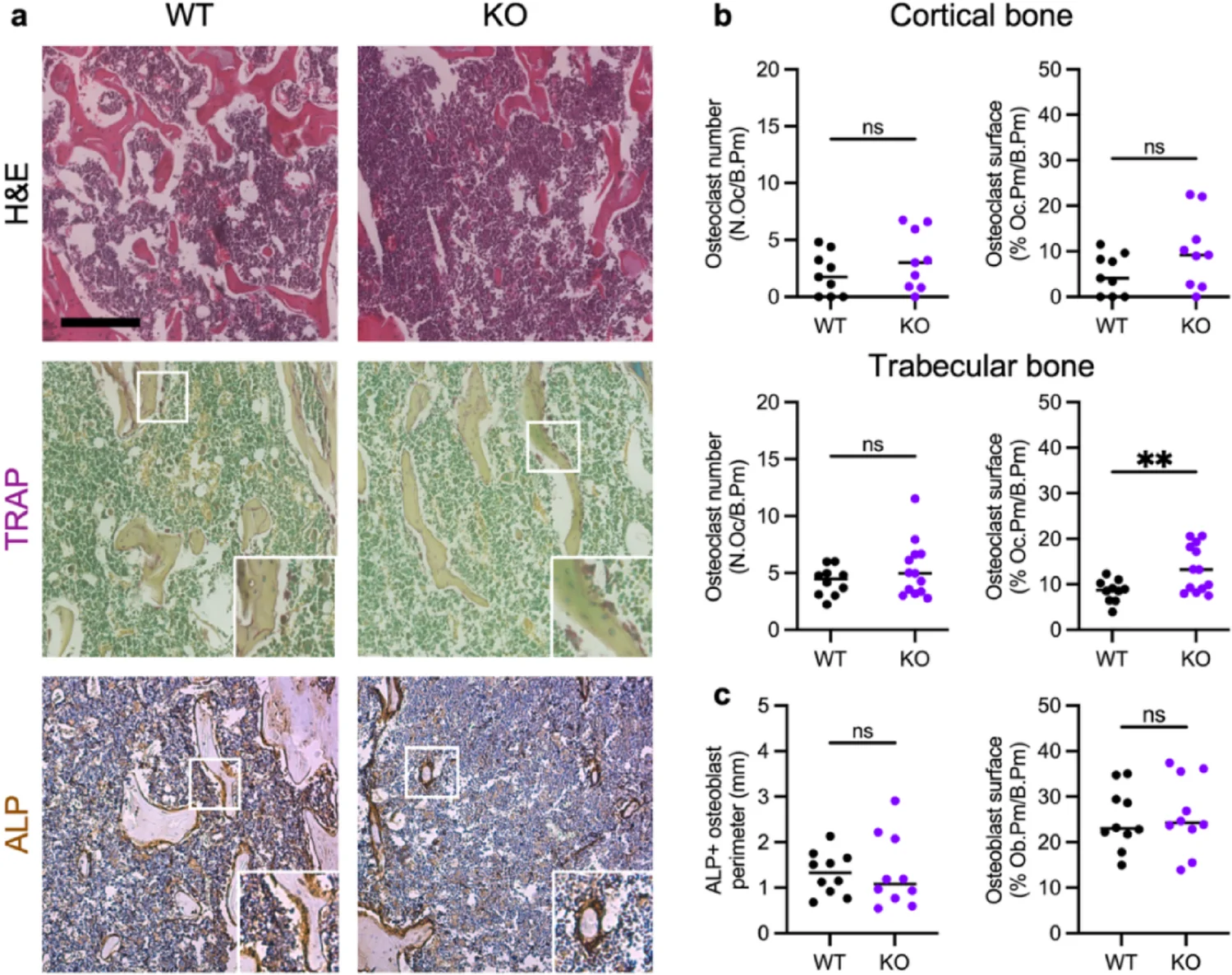
Increased trabecular osteoclast surface area by histological analysis of bone cells in Cxcr2 WT and KO proximal tibia. **a** Representative images of H&E (top), tartrate-resistant acid phosphatase (TRAP) staining (middle), and alkaline phosphatase (ALP) immunohistochemistry (bottom) of Cxcr2 WT and KO tibia. Scale bar = 200 μm. **b** Quantification of TRAP + osteoclast number and surface (% Oc.Pm) per cortical (top) and trabecular (bottom) bone perimeter. **c** Quantification of ALP + bone perimeter and osteoblast surface (%Ob.Pm) per trabecular bone perimeter. Lines indicate median. Statistical analysis of WT versus KO by Welch’s *t*-test. Significance levels for differences are indicated: * *p* < 0.05, **p < 0.01, ***p < 0.001, ns: not significant (*p* ≥ 0.05). *N* = 8–13 bone samples per genotype

The loss of trabecular bone density and alterations in bone morphology that were noted by µCT in Cxcr2 KO bones compared to WT bones were also detectable by H&E staining (Fig. 6a). We used histological examination of osteoclasts (TRAP) and osteoblasts (alkaline phosphatase, ALP) to evaluate further the bone remodeling activity in Cxcr2 WT and KO bones.

We used TRAP staining to identify and analyze osteoclasts on the cortical and trabecular bone surfaces of female Cxcr2 WT and KO proximal tibiae extending 1 mm from the growth plate (Fig. 6b). Few osteoclasts were seen on the cortical bone surface and did not differ in number or area between WT and KO mice. The total surface area of trabecular bone did not differ significantly by genotype; the average total trabecular bone perimeter (B.Pm) measured was 4.61 mm in WT and 3.80 mm in KO bone sections (data not shown). In trabecular bone, osteoclast number (N.Oc/B.Pm) was not different between WT and KO mice (Fig. 6b). However, the osteoclast surface area (% Oc.Pm/B.Pm) increased significantly in Cxcr2 KO bone compared to in WT bone (Fig. 6b). Thus, osteoclast length in Cxcr2 KO trabecular bone significantly increased (*P* = 0.0021), covering 26.23 ± 5.39 μm of bone surface on average, versus an average osteoclast size of 20.22 ± 3.03 μm in WT bone (data not shown). While we did not directly analyze the number of nuclei per osteoclast to address precursor number or fusion specifically, a larger osteoclast area is associated with increased multinucleation and resorption capacity [31–33]. This is consistent with the elevated levels of CTX-1 and the observation of decreased trabecular thickness by µCT and implicates Cxcr2 in regulating osteoclast differentiation and/or activity.

To assess the impact of Cxcr2 deletion on bone formation, we used alkaline phosphatase (ALP) IHC staining to quantify osteoblasts on the trabecular bone surface in Cxcr2 WT and KO tibias (Fig. 6c). Osteoblasts were quantified by analyzing the ALP-positive (ALP+) surface area in tibial sections in the trabecular region extending 1 mm distal from the tibial growth plate. The ALP + surface perimeter and osteoblast surface (% bone perimeter) did not significantly differ between Cxcr2 WT and KO mice. Specifically, the total osteoblast surface area in Cxcr2 KO mice was 26.04 ± 8.18% of the bone perimeter, whereas the wild-type mice exhibited an average of 25.06 ± 6.73% (*P* = 0.77). This indicates that Cxcr2 deficiency does not significantly alter the proportion of the bone surface covered by osteoblasts in the tibia, which is consistent with the unchanged levels of P1NP in Cxcr2 KO versus WT serum (Fig. 2b).

Together, these data indicate that Cxcr2 is not required for the maintenance of osteoblast surface area, while Cxcr2 deficiency disrupts the balance of bone remodeling to favor osteoclast-mediated bone resorption by enhancing osteoclast size and activity [33].

### The Number of Megakaryocytes in Bone Decreases by Cxcr2 Deficiency

**Fig. 7:**
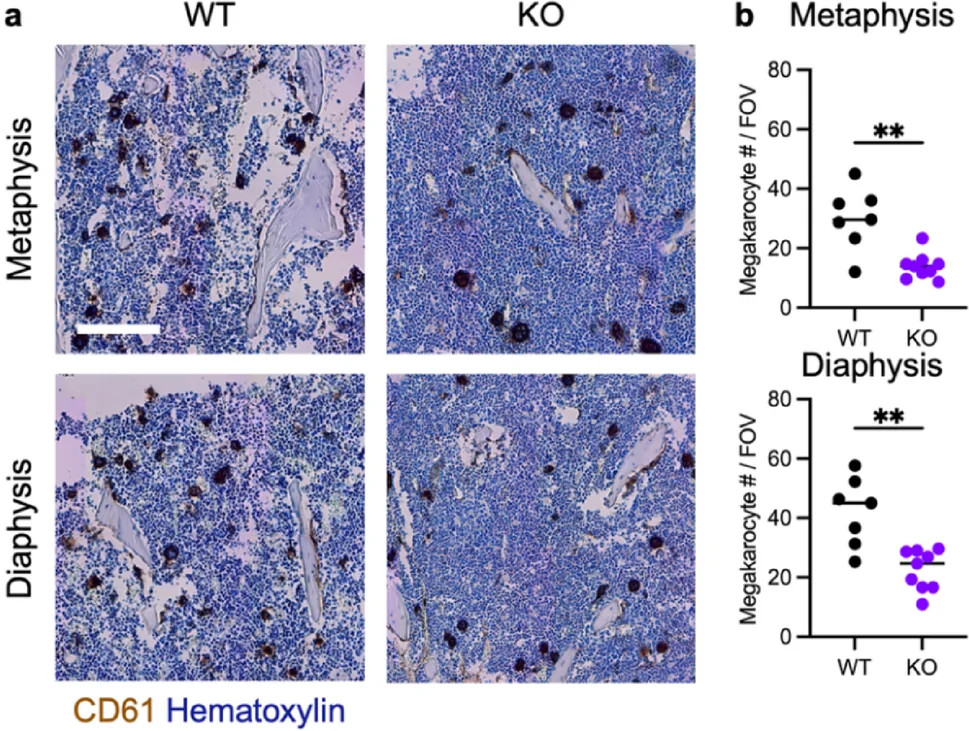
Histological analysis of megakaryocytes in Cxcr2 WT and KO tibia. **a** Representative images of immunohistochemistry for CD61 + megakaryocytes in metaphysis (top) and diaphysis (bottom) of Cxcr2 WT and KO tibia. Scale bar = 200 μm. **b** Quantification of CD61 + megakaryocyte number counted in bone marrow in metaphysis (top) and diaphysis (bottom). Lines indicate median. Statistical analysis used Welch’s *t*-test. Significance levels for differences are indicated: * *p* < 0.05, **p < 0.01, ***p < 0.001, ns: not significant (*p* ≥ 0.05). *N* = 7–9 bone samples per genotype

During histological examination by H&E of Cxcr2 WT and KO bone marrow, we saw a notable decrease in megakaryocytes in Cxcr2 KO bones. These differences were quantified by immunohistochemistry staining of the megakaryocyte cell surface marker CD61 to analyze megakaryocyte number (Fig. 7a). Indeed, bones from Cxcr2 KO mice had significantly fewer megakaryocytes in the metaphysis and diaphysis of the tibia compared to WT bones (Fig. 7b). Specifically, the average megakaryocyte counts in the metaphysis were 30 ± 10 megakaryocytes in WT and 14 ± 4 megakaryocytes in Cxcr2 KO mice per 500 µm^2^ field of view (*P* = 0.0009). In the diaphysis, the average number was 42 ± 12 megakaryocytes in WT mice and 23 ± 7 megakaryocytes in Cxcr2 KO mice per field of view (*P* = 0.0008).

### Increased Neutrophils and RBC Heterogeneity in the Serum of Cxcr2 KO Mice

**Fig. 8:**
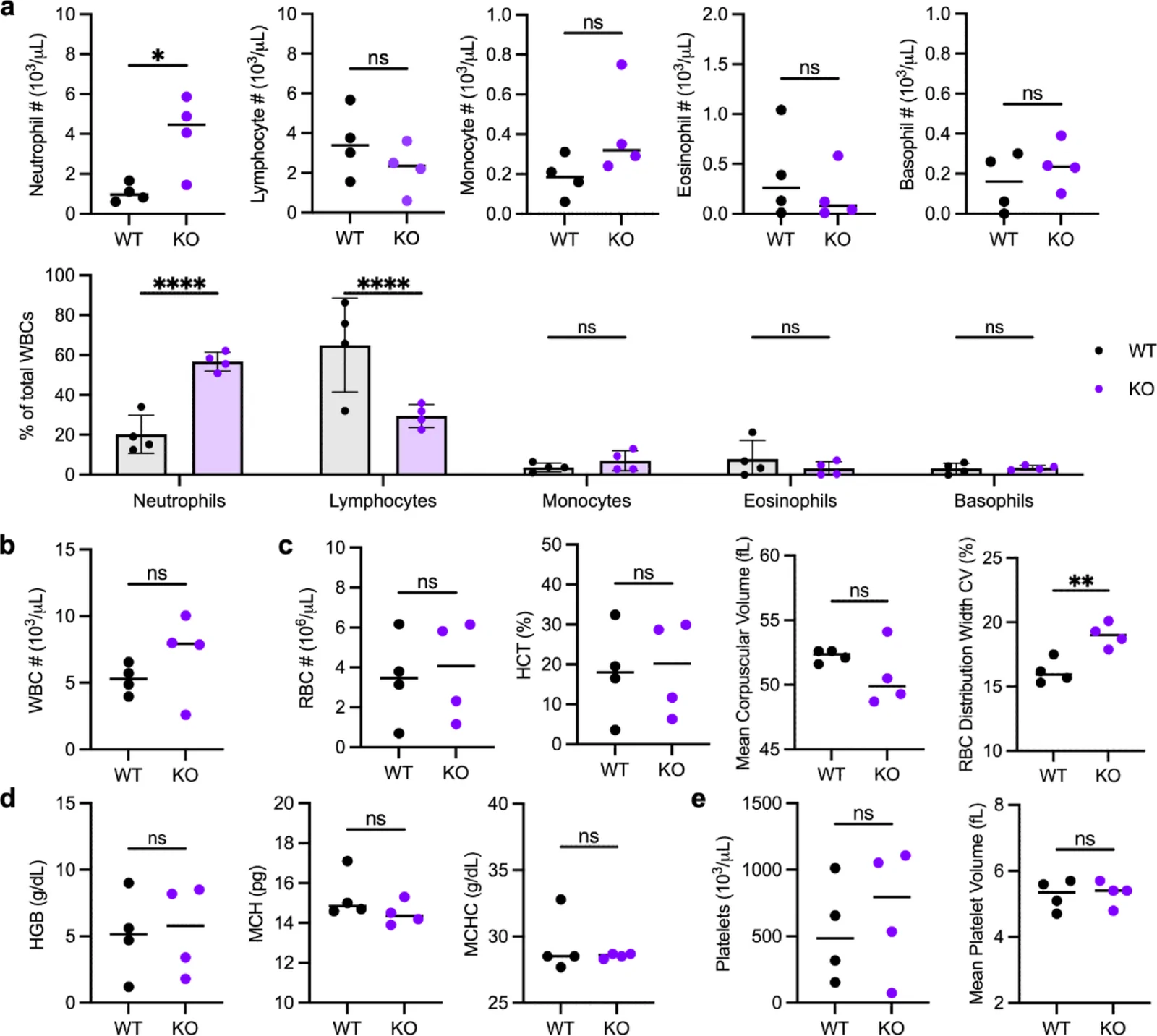
Cxcr2 deletion alters the immune microenvironment. Complete blood count with differential analysis via Element HT5 Hematology Analyzer of cardiac blood from Cxcr2 WT and KO mice. **a** Quantification of count (Top) and percent of total white blood cells (WBCs) (Bottom) of neutrophils, lymphocytes, monocytes, basophils, and eosinophils. Percentages were compared by two-way ANOVA and expressed as mean ± standard deviation. Quantification of **b** total white blood cell (WBC) count and **c** red blood cell (RBC) count, hematocrit (HCT) (% red blood cells of total cells), mean corpuscular volume, and RBC width distribution (percent coefficient of variation). Quantification of **d** hemoglobin parameters, including total hemoglobin (HGB, g/dL), mean corpuscular hemoglobin (MCH), and mean corpuscular hemoglobin concentration (MCHC); and **e** platelet count and volume. Lines depict the median. Statistical analysis of WT vs. KO by Welch’s t-test. Significance levels for differences are indicated: * *p* < 0.05, **p < 0.01, ***p < 0.001, ns: not significant (*p* ≥ 0.05). *N* = 4 per genotype

Cxcr2 is a known critical regulator of neutrophil homeostasis. To determine the impact of Cxcr2 deletion on the systemic immune environment, we analyzed the circulating immune cell profiles in Cxcr2 WT and KO mice using complete blood count with differential analysis via Element HT5 hematology analyzer (Fig. 8).

Consistent with previous reports of granulocyte expansion in Cxcr2 KO mice [18], the circulating neutrophil population expanded significantly in Cxcr2 KO (FVB/N) mice compared to WT mice (Fig. 8a). Specifically, the average neutrophil count in WT animals was 1.05 × 10^3^/µL, compared to 4.06 × 10^3^/µL in KO mice. Similarly, the percentage of neutrophils of total white blood cells was 20.25% in WT mice and 56.75% in Cxcr2 KO mice.

While lymphocyte numbers were unchanged with Cxcr2 deletion (3.50 ± 1.71 × 10^3^/µL in WT compared to 2.23 ± 1.25 × 10^3^/µL in KO), the expanded neutrophil population coincided with a significant decrease in lymphocyte percentage of total white blood cells, from 64.98% in WT mice to only 29.48% in Cxcr2 KO mice. Monocytes, basophils, and eosinophils were unaffected by Cxcr2 deficiency, and there was no difference in total white blood cell counts (Fig. 8a and b).

Red blood cell count, percent, and size as well as hemoglobin concentration were unchanged by Cxcr2 deletion (Fig. 8c and d). However, the RBC width distribution significantly increased in Cxcr2 KO animals (Fig. 8c), indicating anisocytosis, or increased variation in red blood cell size.

Megakaryocytes are precursor cells of platelets. Despite the decreased megakaryocytes observed in KO bone marrow, platelet count and volume were normal in Cxcr2 KO blood (Fig. 8e).

Together, these blood cell data and the serum cytokine analyses (Fig. 2) confirm roles for Cxcr2 in regulating granulocyte populations that may ultimately regulate bone remodeling.

## Discussion

Our study underscores a complex role for Cxcr2 in bone resorption regulation, possibly mediated by altered immune composition and inflammation. First, we characterized the developmental requirements for Cxcr2 in mouse bone structure and biomechanics in Cxcr2 KO mice that we had backcrossed onto the FVB/N background. Body weight, body fat, and overall survival decreased in Cxcr2 KO mice compared to WT mice. In addition, Cxcr2 was required for bone structural integrity. Structural evaluation of the Cxcr2 KO bones revealed a significantly lower trabecular bone volume, marked by thinner trabeculae, compared to WT bones. Cortical bone area and geometric cross-sectional properties also decreased with Cxcr2 deficiency. Subsequent mechanical testing demonstrated that bone strength decreased in Cxcr2 KO mice. At the same time, the bone mineralization was comparable between Cxcr2 KO and WT femurs. These data together suggest that the altered bone mechanical properties are not due to a defect in osteoblast function or mineral homeostasis but instead are caused by decreased bone formation or increased resorption rates.

To examine potential drivers of these architectural defects, we evaluated osteoclast and osteoblast numbers. While osteoclast number was unchanged, the increased osteoclast length, trabecular osteoclast surface area, and CTX-1 levels in Cxcr2 KO bones strongly suggest that bone remodeling was imbalanced toward increased bone resorption due to increased osteoclast activity following Cxcr2 deficiency. This may indicate an increased fusion of precursor cells or cell spreading to increase osteoclast activity at the trabecular bone surface. Histological and hematological analysis further identified immune cell roles for Cxcr2. Megakaryocyte number significantly decreased in Cxcr2 KO mice, though platelet production was unaffected. Neutrophil expansion, anisocytosis, and elevated proinflammatory cytokine signaling significantly accumulated in Cxcr2 KO mice. These changes in the immune cell composition highlight the requirement for Cxcr2 in inflammatory responses and hematological parameters.

Our findings that Cxcr2 deficiency promotes increased bone resorption activity are intriguing and somewhat surprising, given that Cxcr2 and its ligands, which include Cxcl1, 2, and 5, have established roles in promoting osteoclast recruitment, differentiation, and activity. RAW264.7 cells, which are derived from murine myeloid monocytic precursor cells, increase Cxcr2 expression during their differentiation to osteoclasts [14, 34]. Cxcl1, an osteoblast target gene of parathyroid hormone signaling, attracts osteoclast precursor cell populations to the bone niche [13], and Cxcl1 and Cxcl2 expressed in bone marrow adipose tissue induce osteoclast formation [9]. Furthermore, Cxcl5 is associated with high bone turnover in Paget’s disease via RANKL upregulation in pre-osteoblasts, leading to osteoclast maturation and bone resorption [35]. Transgenic mice expressing human IL-8/Cxcl8, which is associated with breast cancer metastasis and osteolysis, have significantly elevated osteoclastogenesis and bone resorption activity in vivo, resulting in low bone volume fraction and decreased trabecular number [36]. Based on these findings, Cxcr2 deletion might be expected to decrease osteoclast activity and bone resorption. However, in contrast, our results revealed increased bone resorption activity and loss of trabecular bone structure in the absence of Cxcr2. The decreased trabecular bone volume fraction and bone mechanical integrity observed with Cxcr2 deficiency in our study are consistent with the bones reported for Cxcr2 KO (BALB/c) [22]. This suggests that Cxcr2 may have a role in suppressing excessive osteoclast activity or in balancing the bone remodeling process, rather than acting directly as an osteoclast activation switch.

The significant immune alterations we saw in Cxcr2 KO mice may contribute to the increased bone resorption. Many of the elevated cytokines are detected during pathogen-induced cytokine storm signaling, which is consistent with the impaired ability to resolve inflammatory responses to environmental pathogens of Cxcr2 KO mice (C57BL/6 [19] and BALB/c [37]). Significantly, proinflammatory cytokines such as G-CSF, CXCL1, TNFα, IL-6, and IL-17, which are known to promote osteoclast activity [38–40], were elevated in Cxcr2 KO serum. These cytokines could exacerbate osteoclast-mediated bone resorption in the absence of Cxcr2, like the pathophysiology and inflammatory bone loss seen in leukocyte adhesion deficiency (LAD)-associated periodontitis, where defective neutrophil recruitment to sites of microbial infection leads to unchecked IL-17 signaling and associated bone resorption [41]. Similarly, the defective neutrophil trafficking observed in Cxcr2-deficient mice may trigger an exaggerated and unresolved inflammatory response that contributes to pathological bone loss. This may help to explain why cortical osteoclasts were not affected in Cxcr2 KO mice, as trabecular osteoclasts are more responsive to local signals in the bone marrow due to the dynamic remodeling of trabecular bone [42]. While RANKL and OPG were both reduced in Cxcr2 KO bone conditioned media, the unchanged ratio supports the role of this inflammatory state, in addition to the direct effect on bone cells, in the altered bone structure of Cxcr2 KO mice. Thus, Cxcr2 may behave as a regulatory knob, fine-tuning osteoclast behavior and modulating the immune and stromal environment to maintain bone remodeling homeostasis.

Environmental factors and pathogen exposure may further contribute to and complicate some of the phenotypes seen in Cxcr2 KO (FVB/N) mice. Our pathogen screening identified the presence of several pathogens, including *Helicobacter ganmani*,* Rodentibacter heylii*, and *Entamoeba muris*, in both WT and Cxcr2 KO animals, suggesting that pathogen-induced inflammation could have amplified the proinflammatory state observed. Importantly, previous studies using Cxcr2 KO (C57BL/6) mice that were rederived in a germ-free condition showed no difference in peripheral blood neutrophils compared to WT animals in the same conditions, but contrasted with the Cxcr2 KO animals bred in specific pathogen-free conditions (like our vivarium), which had significantly elevated neutrophil counts [43]. This study demonstrated that environmental pathogens are required for the observed neutrophilia and may contribute to the release of inflammatory cytokines and subsequent neutrophil production. In addition, exposure to bystander infections in conventional housing ultimately impaired the reproductive ability and development of reproductive organs in Cxcr2 KO mice but were ameliorated in specific and opportunistic pathogen-free animals [44]. This suggests that the interaction between environmental factors and immune dysregulation is likely Cxcr2-dependent and may play a crucial role in other phenotypes, including in the observed bone defects.

In addition to altered cytokine signaling and neutrophilia, megakaryocyte numbers also significantly decreased in Cxcr2 KO mice. This is consistent with previous findings that Cxcr2 deletion in the hematopoietic compartment or treatment with the Cxcr2 inhibitor Repertaxin significantly decreases megakaryocyte number in bone in a mouse fibrosis model [45]. Growing evidence implicates Cxcr2 signaling in regulating megakaryocyte differentiation in myeloproliferative disorders and myelofibrosis [45–47]. Megakaryocytes also regulate bone mass by promoting osteoblastogenesis, osteoblast proliferation, and maintenance of hematopoietic stem cell niches [48, 49]. Thus, their decreased numbers may further exacerbate the hematopoietic and immune dysregulation and disrupted remodeling homeostasis in the bone marrow. Additionally, Cxcr2 KO (BALB/c) mice in conventional housing exhibited altered serum hormone levels, such as decreased progesterone [44], a hormone that promotes osteoblastic activity and bone formation. Because our study did not examine hormone levels in Cxcr2 KO (FVB/N) mice, we cannot rule out the contribution of systemic hormonal changes to the impaired bone structure in these animals.

Laboratory mouse strains have known genomic and phenotypic differences that may influence bone structure. For example, C57BL/6 and FVB/N mouse strains exhibit differences in cortical and trabecular bone parameters and marrow adiposity [50]. Furthermore, crossing C57BL/6 mice with BALB/c or FVB/N strains alters basal cytokine levels, which may influence the utility of transgenic animals as disease models [24, 25]. For instance, C57Bl/6 mice backcrossed onto an FVB background have increased G-CSF in serum compared to C57Bl/6 mice. These differences may lead to an altered stromal microenvironment that affects the sensitivity of the osteoclast response to changes in the inflammatory state induced by Cxcr2 deletion. The enhanced G-CSF signaling observed here in Cxcr2 KO FVB mice may be exacerbated by basal cytokine changes and produce larger, more dysregulated osteoclasts and a more severe bone loss phenotype in the FVB background. These findings highlight the overall importance of considering genetic and environmental modifiers in the interpretation of Cxcr2’s roles in bone homeostasis. Differing cytokine expression, immune responses, and bone biology between mouse strains mimic patient heterogeneity and may help to identify regulatory pathways that are modulated by Cxcr2 in human diseases.

A better understanding of the functions of Cxcr2 and its ligands in the physiological bone environment will provide important insight into Cxcr2 signaling in development, wound healing, metastasis, and other pathologies. Overall, our findings provide new insight into the complex role of Cxcr2 in bone homeostasis. Despite the expected pro-osteoclastogenic function of CXC ligands, the deletion of Cxcr2 increased bone resorption, likely via inflammatory cytokine signaling. This study underscores the need for future research to delineate the precise mechanisms by which Cxcr2 regulates bone turnover, particularly in the context of immune interactions in the bone microenvironment. The Cxcr2 KO FVB/N model offers a valuable tool for investigating the interplay between and contributions of immune cells and bone homeostasis.
